# Patterns of Oral Microbiota in Patients with Apical Periodontitis

**DOI:** 10.3390/jcm10122707

**Published:** 2021-06-19

**Authors:** Izabela Korona-Glowniak, Dominika Piatek, Emilia Fornal, Anna Lukowiak, Yuriy Gerasymchuk, Anna Kedziora, Gabriela Bugla-Płoskonska, Ewelina Grywalska, Teresa Bachanek, Anna Malm

**Affiliations:** 1 Department of Pharmaceutical Microbiology, Faculty of Pharmacy, Medical University of Lublin, 20-093 Lublin, Poland; anna.malm@umlub.pl; 2 Department of Conservative Dentistry with Endodontics, Faculty of Medical Dentistry, Medical University of Lublin, 20-093 Lublin, Poland; dominika.piatek@umlub.pl (D.P.); teresa.bachanek@umlub.pl (T.B.); 3 Department of Pathophysiology, Faculty of Medicine, Medical University of Lublin, 20-090 Lublin, Poland; emilia.fornal@umlub.pl; 4 Institute of Low Temperature and Structure Research, Polish Academy of Science, 50-422 Wroclaw, Poland; a.lukowiak@intibs.pl (A.L.); y.gerasymchuk@intibs.pl (Y.G.); 5 Department of Microbiology, Faculty of Biological Sciences, University of Wroclaw, 51-148 Wroclaw, Poland; anna.kedziora@uwr.edu.pl (A.K.); gabriela.bugla-ploskonska@uwr.edu.pl (G.B.-P.); 6 Department of Clinical Immunology, Faculty of Medicine, Medical University of Lublin, 20-093 Lublin, Poland; ewelina.grywalska@gmail.com

**Keywords:** oral microbiome, apical periodontitis, root canal infection, real-time PCR

## Abstract

In this study, microbial diversity of the root canal microbiota related to different endodontic infections was investigated. In total, 45 patients with endo–perio lesions (8 patients), chronic periapical periodontitis (29 patients) and pulp necrosis (8 patients) were recruited. In 19 (42.2%) patients there was secondary infection of root canals. Microbial specimens were collected from root canals of non-vital teeth with or without changes in periapical area visible in X-ray. Then, oral microbiota were detected and identified using the culture method and real-time PCR amplification primers and hydrolysis-probe detection with the 16S rRNA gene as the target. Overall, 1434 species/genes from 41 different genera of 90 various microbial species were retrieved. Of the major reported phyla, Firmicutes (62.9%), Actinobacteria (14.0%), Bacteroidetes (12.1%), Proteobacteria (9.1%) and Fusobacteria (4.2%) were detected. Of the bacterial species, 54.6% were strict anaerobes. *Corynebacterium matruchotii* (*p* = 0.039) was present significantly more frequently in chronic periapical periodontitis. Moreover, the higher values of Decayed, Missing and Filled Permanent Teeth index were positively correlated with relative abundance of *Actinomyces* spp. (*p* = 0.042), *Lactobacillus* spp. (*p* = 0.006), *Propionibacterium* spp. (*p* = 0.024) and *Rothia* spp. (*p* = 0.002). The multivariate analyses revealed differences in total root canal samples, where components that affected grouping of root samples into four main categories were identified. Anaerobic Gram-negative bacteria predominated in root canals of teeth with pulp necrosis and periapical lesions. Facultative anaerobic Gram-positive bacteria predominated in canals with secondary infections. All detected members of mixed population groups that might serve as keystone species contributed to the entire community in its clinical relevance.

## 1. Introduction

The oral cavity is considered as the second most complex microbiota in human body, following the colon. A high diversity of microorganisms, including bacteria, fungi, viruses, archaea and protozoa belongs to oral microbiota. There are approximately 700 species present in the oral cavity, where 54% have been cultivated and named, 14% have been cultivated but are unnamed, and 32% are known only as uncultivated phylotypes [1,2]. The oral bacterial community is dominated by the six major phyla: Firmicutes, Bacteroidetes, Proteobacteria, Actinobacteria, Spirochetes and Fusobacteria. These six phyla account for 94% of the taxa detected. Among all, Firmicutes contribute the most, at 36.7%, followed by Bacteroidetes (17.1%), Protobacteria (17.1%), Actinobacteria (16.6%), Spirochetes (7.9%) and Fusobacteria (5.2%) [3].

Genera of commensal microbiota in the oral cavity that are associated with a healthy symbiotic relationship contribute to oral health by occupying niches, preventing opportunistic pathogen overgrowth, preventing pathogens from producing virulence factors and degrading virulence factors once they are produced by pathogens [4,5]. Inflammatory diseases of the periapical tissues occur as a result of pulpitis. A small number of microorganisms in the subgingival plaque is involved in the pathogenesis of periodontal disease. Bacteria and their metabolic products penetrate the apical periodontium from the carious lesion through the apical openings of the root canals, as well as through the lateral or chamber-periodontium canals and through the pathological gingival pocket. Along with potential pathogens are commensal microbiota [6]. Therefore, observing the presence of significant pathogen populations and the absence of significant commensal bacteria is often characteristic of the initial stages of periapical disease. A huge challenge in endodontic treatment is the complicated structure of the root canal system as well as the unavailability of bacterial biofilm for disinfectants used for root canal irrigation. Untreated or improperly treated pulp inflammation may lead to late complications, such as chronic inflammation of the periapical tissues. Proper procedure during the treatment, including chemo-mechanical preparation of the canals and their tight obturation up to the apical constriction, as well as the use of biological methods of pulp capping and periodontium protection, should save against the occurrence of complications in the form of periapical tissue inflammation [7,8,9]. There is still an open question about the comparative microbial profiles among different endodontic infections. Increased proportions of certain periodontal pathogens in diseased sites imply the diagnostic value of microbial testing.

Periapical diseases such as an inflammatory disease can lead to systemic inflammation. In prolonged and complicated infections, or due to the high risk of systemic spread of infection, root canal sampling for microbiological diagnostics is recommended. The composition of the microbiota of root canals differs in primary and secondary endodontic treatment. Anaerobic Gram-negative rods are commonly isolated organisms in primary infections, whereas in secondary infections the microbiota is dominated by facultatively anaerobic Gram-positive cocci (*Streptococcus* spp., *Enterococcus* spp., *Peptostreptococcus* spp.) and rods (*Actinomyces* spp.). Recently, *Enterococcus faecalis* and *Candida albicans* have often been detected in treatment-resistant infections [10,11,12].

Cultured-dependent techniques leading to the identification of the microbiota in the root canals of teeth with apical periodontitis were conducted through broad-range culture/biochemical methods, by which only cultivable and predominant bacteria were reachable, with the risk of missing keystone species in development of caries and apical periodontitis. Novel microbial detection methods allow for increases in knowledge about microbial species associated with endodontic infections and their roles in the development of such infections [13,14,15]. The aim of this study was to determine the microbial diversity in root canal samples and formulate a comprehensive map of the bacterial profiles related to different types of endodontic infections using real-time PCR (RT-PCR) amplification.

## 2. Materials and Methods

### 2.1. Patient Selection

**Figure 1:**
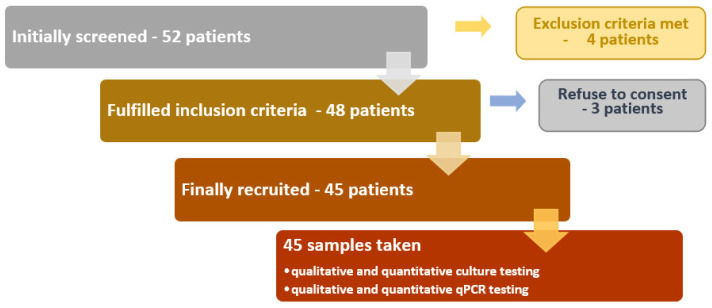
Flowchart of study recruitment.

Adult patients 19–81 years of age with the following conditions were recruited by the Department of Conservative Dentistry with Endodontics, Medical University of Lublin, Poland: pulp necrosis, chronic inflammation of the periapical tissues, indication for re-treatment of root canal (loss of tooth crown filling lasting longer than 48 h, occurrence of periodic pain, discomfort during percussion, presence of sinus tract (fistula), changes in the X-ray image of periapical tissues that have not healed within 2 years of treatment, improperly filled canals in the absence of changes in the periapical tissues when the tooth is intended for prosthetic treatment) or endo–perio lesions (with non-vital pulp). The criteria for exclusion from the study included pregnant, lactating, patients undergoing photodynamic therapy, patients who received antibiotic therapy within the last 3 months or who have immunosuppressive treatment. Patients with over-filling or perforation were also excluded (Figure 1). The same practitioner performed this study in the Department of Conservative Dentistry with Endodontics.

The protocol was reviewed and approved by the Bioethics Committee of the Medical University of Lublin (KE-0254/348/2015) and performed in compliance with Helsinki declaration. Written informed consent was taken from each patient.

Due to the variety of clinical conditions, patients were assigned to three groups:

1. Patients with endo-perio lesions, because it is a specific condition in which we observe both changes in the tooth pulp and changes in the periodontium; in our cases, the changes were very advanced, which made it impossible to establish the primary cause of the observed condition;
2. This group includes patients with pathological conditions of the pulp and periapical tissues, resulting in changes in the X-ray image of the periapical tissues (this includes patients diagnosed with chronic apical periodontitis and exacerbated chronic apical abscess);
3. This group includes patients with pathological conditions of the pulp and periapical tissues without changes in the periapical tissue X-ray image (patients with pulp necrosis and patients qualified for repeated root canal treatment without changes in X-ray, e.g., due to the loss of filling for more than 2 days).

A dental examination was performed, during which the number of teeth with caries, fillings and teeth lost as a result of the carious process was recorded. On this basis, the DMFT (Decayed, Missing, and Filled Permanent Teeth) index was calculated. Teeth were classified for individual components of the index in accordance with the WHO guidelines [16]. In addition, the oral hygiene status was determined based on the API (Approximal Plaque Index) and the periodontium condition using the CPITN (Community Periodontal Index of Treatment Needs) index was calculated according to the WHO recommendations [16]. API was measured with usage of a periodontal probe that was gently placed through the approximal spaces. The first and third quadrants were assessed from the oral aspect, and the second and fourth quadrants from the buccal aspect. Any presence of plaque was noted as a positive result (plus in diagram). The percentage of positive results from all examined sites was counted. Based on the results, API was determined according to the following scheme: (1) <25%—optimal hygiene; (2) 39–25%—rather good; (3) 69–40%—average and (4) 100–70%—poor oral hygiene [17].

### 2.2. Sampling Procedure

The selected for study teeth were subjected to disinfection procedure with the following steps: (i) oral cavity disinfection for 30 s with mouth rinse containing 0.2% chlorhexidine solution; (ii) rubber dam placement with clamp on treated tooth; (iii) rubber dam and tooth structure disinfection with 3% hydrogen peroxide and 2% chlorhexidine; (iv) cavity preparation with access to pulp chamber; (v) pulp chamber disinfection with 2% chlorhexidine placed with cotton ball for 30 s, rinsing with sterile saline solution [18].

At that point, after pulp chamber disinfection, microbiological samples were collected from pulp chambers with sterile paper point size #20 ISO. Then, a first file (FF) ISO size #15 (type S-file) was introduced to the root canal to loosen the biofilm from the root canal walls. Careful introduction of sterile saline solution into the canal was performed with a syringe and endodontic needle, taking care that the root canal would not be overfilled. A sample from the root canal was collected with paper point size #20. Specimens were collected in vials containing 1 mL of Schaedler transport medium (BioMerieux, Marcy l’Etoile, France) and sent immediately to the Department of Pharmaceutical Microbiology, Medical University of Lublin.

### 2.3. Microbial Investigation

Samples were dispersed with a vortex, and 10-fold serial dilutions were made in Schaedler broth (BioMerieux, Marcy l’Etoile, France). Eventually, 0.1 mL from each dilution was placed on Wilkins–Chalgren agar. The plates were incubated for 7 days at 35 °C under anaerobic conditions (80% N_2_, 10% CO_2_, 10% H_2_). After incubation, colony forming units (CFU) were counted. Bacteria were identified with the use of standard bacteriologic methods. Gram-staining was performed on the pure isolates of root canal, and the microbial species were preliminary characterized based on their colony features (size, color, shape, surface and hemolysis). Conventional biochemical tests were performed for identification using an analytical profile index (Vitek 2 Compact, bioMerieux, Marcy l’Etoile, France).

### 2.4. Real-Time PCR Analysis

The root canal samples were stored at −70 °C until RT-PCR could be performed. DNA from root canal samples were extracted using Genomic DNA purification with Nucleo spin (Marchery Nagel, Dueren, Germany) according to the manufacturer’s instructions and analyzed with the Oral Disease Microbial DNA qPCR Array (Qiagen, Germantown, MD, USA). Real-Time PCR assays were performed (Light Cycler 96, Roche, Basel, Switzerland) using the 16S rRNA gene as the target and using PCR amplification primers and hydrolysis-probe detection, which increases the specificity of each assay. Each microbial DNA qPCR array plate analyzed one sample for 93 species (NCBI Tax ID)/gene at a time. Pan-bacteria assays that detect a broad range of bacterial species were included to serve as positive controls for the presence of bacterial DNA. For relative profiling applications, host genomic DNA and overall bacterial load were measured. Inclusion of these analyses allows the user to normalize sample input using ΔΔCT.

### 2.5. Statistical Analysis

The statistical analysis was performed with Tibco Statistica 13.3 (StatSoft, Palo Alto, CA, USA). The values of the parameters were presented as medians, minimum and maximum value. The normal distribution of continuous variables was tested using Shapiro–Wilk test. The Mann–Whitney U-test was used for independent variable comparisons. Kruskal–Wallis ANOVA and multiple comparisons of mean ranks (as post hoc analysis) were applied for the analysis of differences between more than two groups. The power and direction of association between pairs of continuous variables (studied groups) were determined using Spearman’s coefficient of rank correlation. The distributions of discrete variables in groups were compared with the Pearson’s Chi-square test or the Fisher’s exact test. The multivariate data analyses were carried out using the SIMCA 16 (v16.0.2, Umetrics, Sweden). Relative bacterial species abundance in root samples were calculated according to the real-time PCR data analyzing protocol [19]. Principal component analysis (PCA) was used for identifying similarities and differences between analyzed samples. Data were scaled to unit variance and centered. Hierarchical Cluster Analysis (HCA) and partial last square discriminant analysis model (PLS-DA) were used for root sample classification and predictions. A final significance test was performed with the use of a CV-ANOVA (analysis of variance of the cross-validated residuals) test to verify the model’s validity. The model was only considered to be valid if the permutation test and the CV-ANOVA test were satisfied at the same time. In order to determine the sensitivity and specificity of the established models, receiver operating characteristic curves (ROC) of true positive rates were plotted as a function of false positive rates.

## 3. Results

### 3.1. Clinical Features

Out of 45 patients with an age range of 19–81 years (mean 49.4 ± 18.3), 23 were males and 22 were females. In selected patients, endo–perio lesions (8 patients), chronic periapical periodontitis (29 patients), and pulp necrosis (8 patients) were diagnosed. In 19 patients, it was a secondary infection of root canals. The demographic and clinical measurements for all participants are listed in Table 1. No significant differences were found between parameters of full mouth examination in patients with diagnosed endodontic diseases.

### 3.2. Microbiota of the Root Canals by Culture and Real-Time PCR

Using the microbiological culture method, 286 cultivable isolates, representing 12 different genera and 14 distinct microbial species, were retrieved from 45 root canal samples. In these specimens, the number of microbial species ranged from two to seven per sample. More than 19% of isolates were non-identified. The number of cultivable bacteria ranged from 1.0 × 10^1^ to 1.47 × 10^7^ (median 5.2 × 10^4^) CFU/mL. In samples collected from patients with endo–perio lesions, chronic periapical periodontitis and pulp necrosis of total bacterial count had no statistically significant difference (*p* = 0.97). No significant differences were found in total bacterial count of patients with primary and secondary infection (*p* = 0.70).

**Figure 2:**
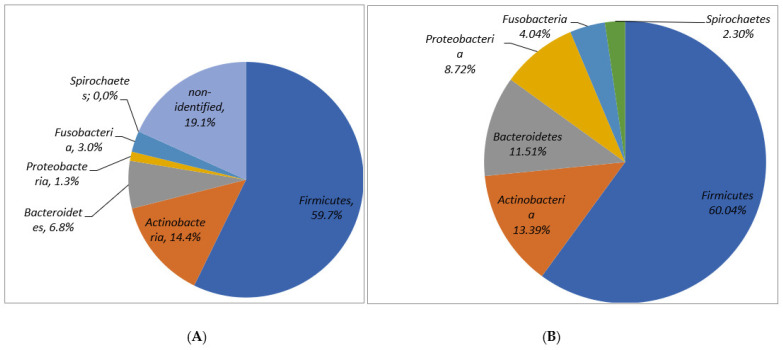
Proportion of occurrence among the root apex samples from teeth with apical periodontitis obtained by culture (**A**) and molecular (**B**) methods.

In molecular analysis with real-time PCR, from the 45 patients studied, 1434 species/genes from 41 different genera of 90 various microbial species were retrieved. In one sample 5–61 (mean 31.8 ± 15.3) species/genes were detected. Of the major reported phyla, Firmicutes (60.04%), Actinobacteria (13.4%), Bacteroidetes (11.51%), Proteobacteria (8.72%) and Fusobacteria (4.04%) were detected. Despite the non-identified number of isolates in the conventional culture method, the percentage of identified phyla in culture was comparable to the results found with use of the molecular method (Figure 2).

### 3.3. Microbiota Profiles in Relation to Endodontic Infections

**Figure 3:**
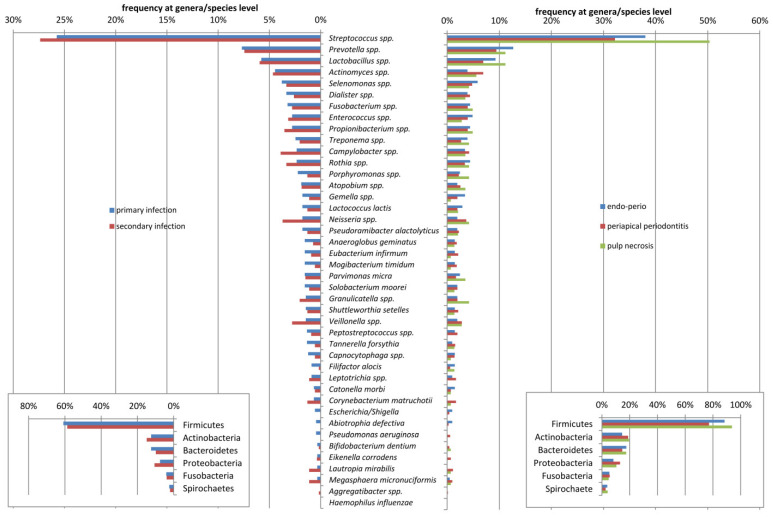
The distribution of microorganisms in the root canal samples from patients with primary and secondary endodontic infections and with different entities of apical periodontitis obtained by molecular methods.

A variety of species was detected in patients with different diagnoses, although no statistical significance was observed. Mean numbers of bacterial species in one sample were 35.4 ± 13.8 (range 17–54), 32.3 ± 15.7 (range 5–61) and 26.9 ± 16.1 (range 6–50) observed in endo–perio, periapical periodontitis and pulp necrosis patients, respectively. The distribution of microorganisms in the root canal samples from patients with primary and secondary endodontic infections and with different entities of apical periodontitis obtained by molecular methods are presented in Figure 3.

The prevalence of Socransky red complex pathogens in patients, namely *P. gingivalis*, *T. forsythia* and *T. denticola*, was 22.2%, 33.3% and 24.4%, respectively. The most prevalent isolated species, except *Streptococcus* spp. (*S. infantis*, *S. sanguinis*, *S. pneumoniae, S. mitis*, *S. mutans*, *S. salivarius*), being the very most prevalent (27–38/45, 60–84.4%), were *Propionibacterium acnes* (41/45, 91%), *Lactobacillus paracasei/casei/zeae* (32/45, 71.1%), *E. faecalis* (31/45, 68.9%), *Rothia aeria/dentocariosa* (28/45, 62.2%), *L. gasseri* (27/45, 60%) and *Parvimonas micra* (22/45, 48.9%). Gram-negative cocci and bacilli were also detected, including the *Fusobacterium nucleatum* (36/45, 80%), *Prevotella nigrescens* (27/45, 60%), *Dialister invisus* (26/45, 57.8%), *Prevotella oris* (24/45, 53.3%), *Pseudoramibacter alactolyticus* (23/45, 51.1%), *Selenomonas sputigena* (23/45, 51.1%) and *Veillonella parvula* (23/45, 51.1%).

**Figure 4:**
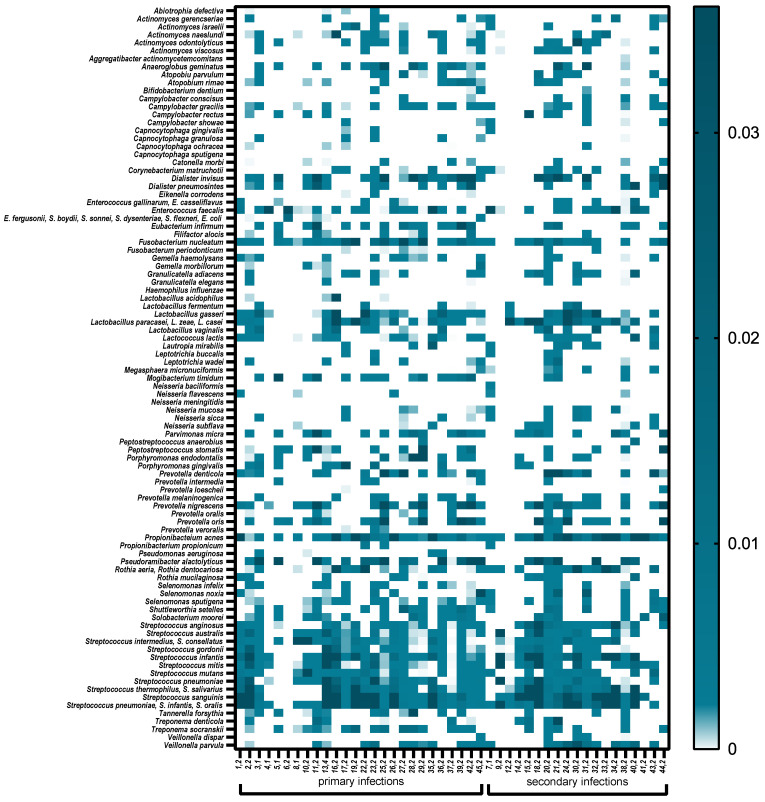
Heat map showing the relative abundance of species/genera genes across samples.

There were no associations between the frequency of these bacteria and the oral disease. However, the statistical analysis revealed the differences in prevalence of several species in patients with different diagnoses (Table 2). *Capnocytophaga ochracea* (*p* = 0.025), *Gemella haemolysans* (*p* = 0.029), *Neisseria flavescens* (*p* = 0.028), *Prevotella denticola* (*p* = 0.018) and *Rothia mucilanigosa* (*p* = 0.018) were significantly associated with endo–perio lesions, whereas *Corynebacterium matruchotii* (*p* = 0.039) was significantly more frequently present in chronic periapical periodontitis. Interestingly, *Actinomyces naeslundi* (*p* = 0.046), *Anaeroglobus geminatus* (*p* = 0.027), *Filifactor alocis* (*p* = 0.037), *Mogibacterium timidum* (*p* = 0.01), *Streptococcus australis* (*p* = 0.048), *Streptococcus mutans* (*p* = 0.046), *Tannerella forsythia* (*p* = 0.033) *and Treponema socranskii* (*p* = 0.045) were found more frequently in patients with primary infections (Table 2). A total of 57.6% of the bacterial species were strict anaerobes. Anaerobic Gram-negative bacteria predominated in root canals of teeth with pulp necrosis and periapical lesions. Facultative anaerobic Gram-positive bacteria predominated in canals with secondary endodontic infections. Community differences between the two pathologies were also observed at the phylum level. Microbial profiles showing microbial composition and relative abundance of root canal samples are presented in Figure 4.

Clinical parameters were correlated to relative species/genera abundance. It was shown that the higher values of DMFT were positively correlated with *Actinomyces* spp. (R = 0.30, *p* = 0.042), *Lactobacillus* spp. (R = 0.40, *p* = 0.006), *Propionibacterium* spp. (R = 0.34, *p* = 0.024) and *Rothia* spp. (R = 0.45, *p* = 0.002) and negatively with *Escherichia/Shigella* (R = −0.38, *p* = 0.01). Approximal plaque index (API) was positively correlated to a higher abundance of *Anaeroglobus geminatus* (R = 0.32, *p* = 0.32), *Mogibacterium timidum* (R = 0.41, *p* = 0.005) and *Porphyromonas* spp. (R = 0.32, *p* = 0.035).

### 3.4. Bacterial Relationships within the Root Canal Communities

**Figure 5:**
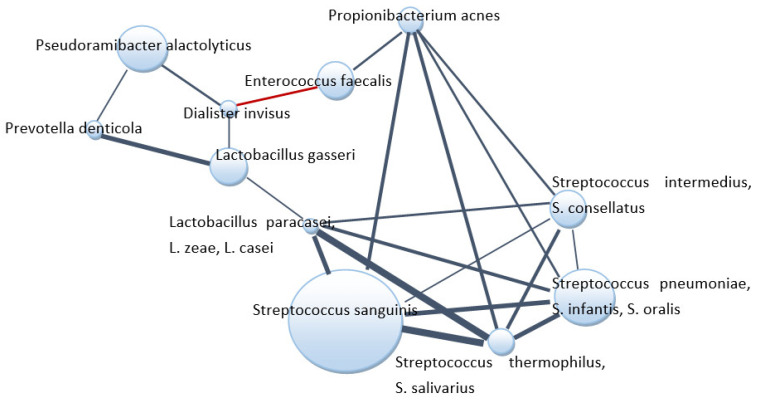
Co-occurrence patterns among most abundant species/genera in root canal samples. The bacteria found in at least eight samples and with a minimal average relative abundance of 1% (considering all root canal samples) were analyzed. The edges represent positive (blue) and negative (red) Spearman correlations (*p* < 0.05). The line thickness is proportional to the absolute value of the Spearman correlation. Node sizes reflect average relative abundance of each species’/genera’s genes.

The co-occurrence of microbial species within the root canal communities was performed by a correlation network analysis to find associations between bacteria (Figure 5). Streptococci were positively correlated with each other, with lactobacilli species and *P. acnes. E. faecalis* negatively correlated with *Dialister invisus* but displayed positive associations with *P. acnes*. *D. invisus* correlated positively with three bacterial species identified.

### 3.5. Prediction of Bacterial Communities Profiles—Multivariate Analysis

**Figure 6:**
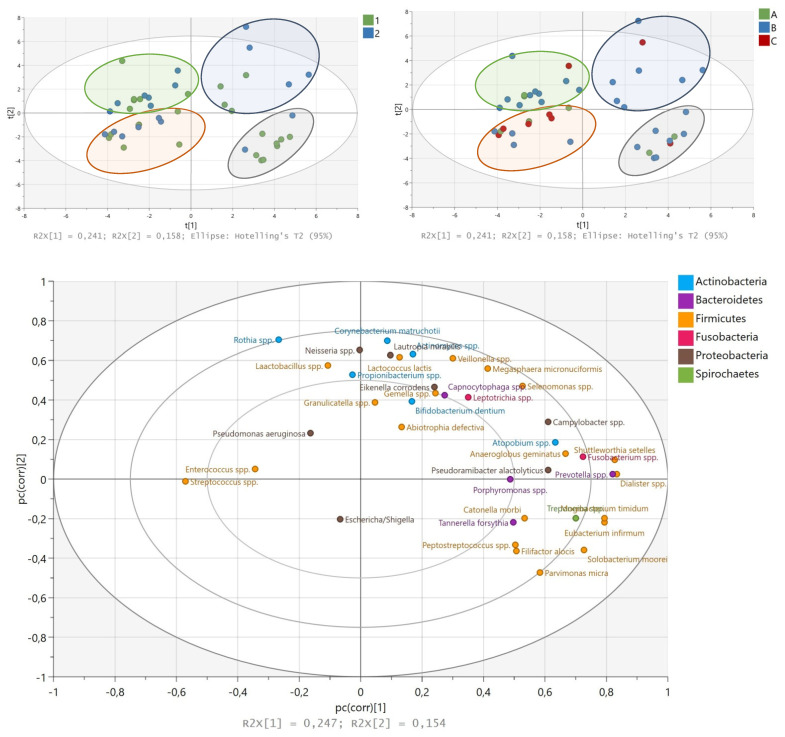
PLS-DA with root canal samples was performed based on the taxonomic profiles at the genus level. Different colors of points in plots were represented for different groups: (**A**) the blue points represent samples from patients with secondary infection, the green points represent samples from patients with primary infection; (**B**) the blue points represent samples from periapical periodontitis patients, the green points represent samples from endo–perio patients, and the red points represent samples from pulp necrosis patients; (**C**) the correlation loadings plot obtained in partial least square-discriminant analysis (PLS-DA) represent bacterial genera included in the analysis.

Principal component analysis (PCA) was initially applied to compare the overall structure of root canal microbiota of all samples using data scaled to UV (Figure 6). The first principal component explained 24.8% of the overall variability among different groups, whereas the second principal component explained 15.5% of the variability. Hierarchical cluster analysis and PLS-DA based on the relative abundance of species/genera revealed a separation of four groups of samples (red group, green group, blue group and orange group, Figure 6) on the basis of the first two principal component (PC) scores. This discrimination was also confirmed by discriminant analysis (PLS-DA). No obvious clustering by pathology was observed for tested root canal samples. As shown in Figure 6A–C, an apparent microbial composition clustering pattern was identified for each separated group. The closer the variable was to the circle, the more it was correlated with the component. The first PC was negatively correlated with *Streptococcus* spp. and *Enterococcus* spp. abundance, and significantly positively correlated with *Shuttelworthia setelles*, *Dialister* spp., *Mogibacterium timidum*, *Prevotella* spp. *Solobacterium moorei*, *Fusobacterium* spp., *Treponema* spp. and *Anaeroglobus geminatus*. The second PC was significantly positively correlated with *Rothia* spp., *Corynebacterium matruchotii, Actinomyces* spp., *Lactococcus lactis, Neisseria* spp., *Propionibacterium* spp. and *Lactobacillus* spp. and in negatively with *Parvimonas micra*.

Mann–Whitney tests showed that the higher relative abundances of *Anaeroglobus geminatus* (*p* = 0.047), *Filifactor alocis* (*p* = 0.037), *Mogibacterium timidum* (*p* = 0.037) and *Tannerella forsythia* (*p* = 0.044) were significantly different between patients with primary and secondary infections. According to PCA, these species along with *Shuttelworthia setelles*, *Parvimonas micra*, *Solobacterium moorei*, *Dialister* spp. *Prevotella* spp., *Pseudoramibacter* spp., *Eubacterium infirmum* and *Treponema* spp. were high in the green group of patients represented mostly by primary infected patients. *Streptococcus* spp., *Rothia* spp. and *Enterococcus* spp. contributed mainly in the red group of patients, which included mostly pulp necrosis patients. *Rothia* spp., *Enterococcus* spp. and *Lactobacillus* spp. were high in the orange group, which contained periapical periodontitis samples. The blue group of patients also consisted of the samples from periapical periodontitis and was enriched by *Actinomyces* spp., *Corynebacterium matruchotii, Megasphera micronuciformis, Lactococcus lactis, Selenomonas* spp, *Capnocytophaga* spp., *Neisseria* spp., *Leptotricha* spp., *Rothia* spp., *Veillonella* spp. and *Propionibacterium* spp.

## 4. Discussion

This study evaluated the microbiota present exclusively in the apical root canal system. Bacteria located in this region are in a strategic position to inflict damage to the host to induce and maintain periapical inflammation. Indeed, culture studies revealed that the large majority of teeth with post-treatment apical periodontitis had bacterial infection in the apical canal system [20,21,22,23]. In this study, microbiota samples from the apical part of the root canal from endodontic infection with different entities were collected. Using the conventional culture method along with the 16S rRNA gene as the target, 93 species/genes were detected, and attention was paid to the bacterial community in the apical part of the root canal to investigate the microbial features with respect to apical periodontitis. To the best of our knowledge, this is first attempt to map the bacterial profiles related to different types of endodontic infections.

The microbial counts and number of taxa involved in the development of endodontic disease depend on geographic location, socioeconomic status and dietary habits [24]. Its composition has been studied by conventional cultural methods for centuries. However, traditional bacterial culture methods permit the culture of a limited portion (<50%) of bacteria [13]. The expanded Human Oral Microbiome Database contains the information of approximately 772 prokaryotic species, where 70% is cultivable, and 30% belong to the uncultivable class of microorganisms [3]. Moreover, out of 70% cultivable species, 57% have already been assigned to their names. In our study, almost 20% of cultivated isolates were non-identified, and isolates obtained with the use of conventional cultures constituted only 15% of species identified using molecular techniques targeting amplification of 16S rRNA. An interesting finding of this work was the similarity of the data achieved by real-time PCR and culture, particularly regarding the number of the taxa detected. It was not surprising that the phylum Firmicutes was most frequently detected, in all pathologies by both methodologies.

Our results revealed a polymicrobial profile of the tested samples, as previously reported [14,25]. Primary infected root canals are untreated canals where microorganisms were able to access and colonize the pulpal tissue and impair its function. As was shown earlier, their microbial profile consists of 10–30 species per canal [15,26]. In our study, a higher number of species in one sample in patients with primary infection in comparison to secondary infection (mean 34.5 ± 14.5 vs. 28.3 ± 16.2, *p* = 0.12) was observed. Bouillaguet et al. [27] noted a significant decrease in bacterial diversity in secondary infection samples compared with primary ones. However, other studies reported an increased diversity in secondary infection samples [28] or no significant difference between the two types of infection [14,29].

It is known that many of the periodontal pathogens are also endodontic pathogens; however, there are few studies dedicated to the investigation of combined endodontic–periodontal lesions [30,31,32]. This study confirmed previous findings regarding the microbial population of root canals, which included species of *Fusobacterium, Peptostreptococcus, Porphyromonas, Prevotella* and *Streptococcus* genera. A higher prevalence of *Streptococcus* spp. in our study could be caused by external contamination of root canals that occurred during sampling, although care was taken to control possible contamination. However, a significantly higher prevalence of *Streptococcus* spp. in pulp necrosis samples (50.3%) compared to endo–perio lesions (50.3% vs. 38.0%, *p* = 0.028) and periapical periodontitis (50.3% vs. 32.2%, *p* < 0.0001) may confirm their impact on disease development.

Facultative anaerobic and Gram-positive bacteria predominated in canals with endodontic treatment failure, which may be due to the increased resistance to instrumentation and to antiseptic agents [33]. In our study, a higher relative abundance of streptococci, lactobacilli and *Propionibacterium* spp. in secondary infected patients was detected. According to Molander et al. [34], Gram-positive facultative and aerotolerant anaerobes, can endure in an inactive, low metabolic state for some time, and when living conditions change due to coronal leakage during or after root canal treatment, bacterial growth can occur.

However, the proportion of certain bacterial taxa, the presence of taxa (phyla and genera) discriminating between primary and secondary apical periodontitis and differences in bacterial diversity between these pathologies varied among studies. In the study of Keskin et al. [29], Proteobacteria was a dominant phylum, whereas in others, including ours, it was much less represented. Similar to the Tzanetakis et al. study [28], it was found that root canal microbiota associated with secondary infection harbored higher levels of Proteobacteria and Actinobacteria than those from patients with primary infection, but these differences did not reach the threshold of statistical significance.

The differences between root canal microbiota in many studies may result from a combination of factors, including differences in patient inclusion/exclusion criteria, tooth treatments or methodologies used to collect the microbiota. In this study, only root canal microbiota collected using paper points inserted into the root canal during the course of an endodontic treatment were used, considering the fact that bacteria in close surrounding of the periapical tissues were more likely to develop apical periodontitis [35,36]. Moreover, the influence of geographic related factors, including environmental contaminants from food or water, have been shown to impact the oral microbiota [28,37].

In our study, each assay was based on PCR amplification of a species-specific genetic region of the relevant microbe. The amplified product is detected using target-specific fluorescent hydrolysis probes, which helps to improve the specificity of the assay. Assays for detection of bacterial species target the 16S rRNA gene and were designed using the GreenGenes database for 16S sequences. The technique used in this study is an easier and quicker method allowing one to detect 93 genera/species specific genes in comparison to the next generation sequencing (NGS) technique. However, the limitation of the study is that some species reported in other studies carried out with the use of NGS technique were not detected. This variety of parameters may render it difficult to compare trends in the composition of the root microbiota associated with apical periodontitis. However, variability between studies using complex NGS workflows is also unavoidable and may occur during the DNA extraction, PCR amplification (the choice of the 16S rRNA gene variable regions to be amplified), sequencing or bioinformatic analysis pipeline. On this account, the same taxa tend to be detected in every endodontic infection, yet with different relative abundances [14,27,28,29].

This study further attempted to analyze the co-presence or exclusion of bacterial species in root canals. *Dialister invisus,* which establishes multiple interactions with other species in apical periodontitis, belongs to the genus frequently identified as a member of the endodontic microbiota of infected root canals [15,27]. Most bacteria in this study created positive correlations; *Propionibacterium acnes*, *Lactobacillus* spp. and different species of *Streptococcus* formed network of interactions. Given the polymicrobial origin of apical periodontitis, central species in the networks of interactions may be considered as possible keystone pathogens in apical periodontitis and account for putative targets for therapeutic interventions [27,38].

In this study, *Enterococcus faecalis* was one of the most frequently identified (68.9%) that is in agreement with other studies that noted the levels of *E. faecalis* ranging from 0% to 90% [29,39,40]. A strong negative correlation between *E. faecalis* and *D. invisus*, frequent species mainly in patients with primary infection, was observed. *E. faecalis* was reported as most abundant in secondary apical periodontitis samples [27] but was not the finding of our study. Interestingly, despite similar average abundance of the species in primary and secondary infections (7.9% vs. 5.7%), the fairly high average abundance was observed in pulp necrosis samples (12.5%) in comparison to endo–perio lesions (0.34%) and periapical periodontitis (7.3%). In this line, a hierarchical cluster analysis showed a clear clustering of the pulp necrosis group according to their content of *E. faecalis*. Whereas some samples exhibited abundances above 90%, others appeared devoid of the species. The limitation of the study is the small number of recruited patients and lack of follow up to compare treatment outcomes with the bacterial profile detected during first sampling after diagnosis.

## 5. Conclusions

Comparison of the microbial populations in different periapical diseases is essential to understand interactions of these bacterial communities and may help to establish appropriate therapeutic procedures for a more predictable outcome of endodontic treatment. Overall, findings from our study demonstrate that all types of investigated endodontic diseases correlated with a highly diverse microbiota. In our study, the multivariate analyses revealed the differences in total root canal samples analyzed with the real-time PCR method, where components that affected grouping of root canal samples into four main categories were identified. It should be pointed out that all detected members of mixed population groups are important, as they might serve as keystone species contributing to the entire community in its clinical relevance.

## Figures and Tables

**Table 1:** Demographic and clinical characteristics of the study population.

Parameter	Endo–Perio Lesions (*n* = 8)	Chronic Periapical Periodontitis (*n* = 29)	Pulp Necrosis (*n* = 8)	*p* Value	Primary Infection (*n* = 26)	Secondary Infection (*n* = 19)	*p* Value
Age (median, range)	54.5 (40–65)	46 (19–80)	70 (24–81)	0.28	46.0 (22–80)	59 (19–81)	0.66
Female (%)	5 (62.5)	12 (41.4)	5 (62.5)	0.4	11 (42.3)	11 (57.9)	0.23
Full mouth examination:							
API (median, range)	3.0 (3–4)	4 (2–4)	3.5 (3–4)	0.82	4.0 Z (3–4)	3.0 (2–4)	0.06
DMFT (median, range)	25 (16–31)	21 (10–32)	25 (10–30)	0.6	22.5 (10–32)	23.0 (10–31)	0.55
D (median, range)	3 (2–13)	5 (1–13)	4.5 (3–9)	0.75	4.5 (1–13)	5.0 (1–13)	0.4
M (median, range)	13 (0–24)	5 (0–28)	12 (0–18)	0.38	8 (0–28)	10.0 (0–18)	0.47
F (median, range)	7 (2–14)	7 (1–18)	7 (6–11)	0.88	5.5 (1–18)	8.0 (5–16)	0
CPI (median, range)	3.5 (2–4)	3.0 (0–4)	2.5 (2–3)	0.06	3.0 (2–4)	2.0 (0–4)	0.02
Secondary infection (%)	0 (0)	13 (44.8)	6 (75.0)	0.009	-	-	-

API, Approximal Plaque Index; DMFT, Decayed, Missing, and Filled Permanent Teeth; CPI, Community Periodontal Index.

**Table 2:** Prevalence of significant bacterial species in patients with different diagnosis.

Prevalence (%)/Relative Bacterial Abundance (Median, Range)	Endo–Perio Lesions (*n* = 8)	Chronic Periapical Periodontitis (*n* = 29)	Pulp Necrosis (*n* = 8)	*p* Value
*Capnocytophaga ochracea*	3 (37.5)	1 (3.45)	1 (12.5)	0.025
*Gemella haemolysans*	5 (62.5)	10 (34.48)	0 (0)	0.029
*Neisseria flavescens*	3 (37.5)	2 (6.9)	0 (0)	0.028
*Prevotella denticola*	7 (87.5)	12 (41.4)	3 (37.5)	0.028
*Rothia mucilanigosa*	5 (62.5)	4 (13.8)	2 (25.0)	0.018
*Corynebacterium matruchotii*	0 (0)	12 (41.38)	1 (12.5)	0.039
Actinobacteria	3.6 × 10^−2^ (1.1 × 10^−5^–23.9 × 10^−2^)	0.8 × 10^−2^ (0–78.5 × 10^−2^)	0.3 × 10^−2^ (0–55.9 × 10^−2^)	0.67
Bacteroidetes	0.5 × 10^−2^ (1.1 × 10^−5^–55.8 × 10^−2^)	1.7 × 10^−2^ (0–77.2 × 10^−2^)	0.5 × 10^−2^ (0–54.9 × 10^−2^)	0.93
Firmicutes	86.0 × 10^−2^ (37.0 × 10^−2^–99.9 × 10^−2^)	55.7 × 10^−2^ (4.1 × 10^−2^–99.9 × 10^−2^)	94.0 × 10^−2^ (29.4 × 10^−2^–29.4 × 10^−1^)	0.034
Fusobacteria	0.15 × 10^−2^ (0–7.1 × 10^−2^)	0.1 × 10^−2^ (0–15.1 × 10^−2^)	0.2 × 10^−2^ (0–4.2 × 10^−2^)	0.91
Proteobacteria	0.01 × 10^−2^ (0.4 × 10^−5^–4.8 × 10^−2^)	2.5 × 10^−2^ (0–94.9 × 10^−2^)	0.9 × 10^−2^ (0–9.7 × 10^−2^)	0.048
Spirochetes	0.9 × 10^−2^ (0–11.9 × 10^−2^)	0 (0–18.6 × 10^−2^)	0.07 × 10^−2^ (0–0.5 × 10^−2^)	0.97
Prevalence (%)/Relative bacterial abundance (median, range)	Primary infection (*n* = 26)	Secondary infection (*n* = 19)		*p* Value
*Actinomyces naeslundi*	13 (50.0)	4 (21.1)		0.046
*Anaeroglobus geminatus*	14 (53.9)	4 (21.1)		0.027
*Filifactor alocis*	8 (30.8)	1 (5.3)		0.037
*Mogibacterium timidum*	14 (53.9)	3 (15.8)		0.0098
*Streptococcus australis*	22 (84.6)	11 (57.9)		0.048
*Streptococcus mutans*	21 (80.8)	10 (52.6)		0.046
*Tannerella forsythia*	12 (46.2)	3 (15.8)		0.033
*Treponema socranskii*	16 (61.5)	6 (31.6)		0.045
Actinobacteria	0.8 × 10^−2^ (0–23.9 × 10^−2^)	2.1 × 10^−2^ (0–78.5 × 10^−2^)		0.13
Bacteroidetes	1.3 × 10^−2^ (0–77.2 × 10^−2^)	0.04 × 10^−2^ (0–34.2 × 10^−2^)		0.25
Firmicutes	67.2 × 10^−2^ (5.3–99.9 × 10^−2^)	73.1 × 10^−2^ (4.1–100 × 10^−2^)		0.67
Fusobacteria	0.2 × 10^−2^ (0–15.1 × 10^−2^)	0.09 × 10^−2^ (0–5.5 × 10^−2^)		0.24
Proteobacteria	1.6 × 10^−2^ (0–85.0 × 10^−2^)	0.6 × 10^−2^ (0–94.9 × 10^−2^)		0.51
Spirochetes	3.4 × 10^−5^ (0–11.1 × 10^−2^)	0 (0–18.6 × 10^−2^)		0.21

## Data Availability

Due to privacy and ethical concerns, the data that support the findings of this study are available on request from the authors [I.K.-G. and D.P.].

## References

[1] Zhang Y., Wang X., Li H., Ni C., Du Z., Yan F. (2018). Human oral microbiota and its modulation for oral health. Biomed. Pharmacother..

[2] Dewhirst F.E., Chen T., Izard J., Paster B.J., Tanner A.C., Yu W.H., Lakshmanan A., Wade W.G. (2010). The human oral microbiome. J. Bacteriol..

[3] Verma D., Garg P.K., Dubey A.K. (2018). Insights into the human oral microbiome. Arch. Microbiol..

[4] Kumar P.S., Mason M.R. (2015). Mouthguards: Does the indigenous microbiome play a role in maintaining oral health?. Front. Cell. Infect. Microbiol..

[5] Zarco M.F., Vess T.J., Ginsburg G.S. (2012). The oral microbiome in health and disease and the potential impact on personalized dental medicine. Oral Dis..

[6] Jenkinson H.F., Lamont R.J. (2005). Oral microbial communities in sickness and in health. Trends Microbiol..

[7] Siqueira J.F. (2001). Aetiology of root canal treatment failure: Why well-treated teeth can fail. Int. Endod. J..

[8] Nair P.N. (2006). On the causes of persistent apical periodontitis: A review. Int. Endod. J..

[9] AlRahabi M.K. (2017). Evaluation of complications of root canal treatment performed by undergraduate dental students. Libyan J. Med..

[10] Gomes B.P., Pinheiro E.T., Gadê-Neto C.R., Sousa E.L., Ferraz C.C., Zaia A.A., Teixeira F.B., Souza-Filho F.J. (2004). Microbiological examination of infected dental root canals. Oral Microbiol. Immunol..

[11] Pinheiro E.T., Gomes B.P., Drucker D.B., Zaia A.A., Ferraz C.C., SouzaFilho F.J. (2004). Antimicrobial susceptibility of *Enterococcus faecalis* isolated from canals of root filled teeth with periapical lesions. Int. Endod. J..

[12] Barbosa-Ribeiro M., De-Jesus-Soares A., Zaia A.A., Ferraz C.C., Almeida J.F., Gomes B.P. (2016). Antimicrobial susceptibility and characterization of virulence genes of Enterococcus faecalis isolates from teeth with failure of the endodontic treatment. J. Endod..

[13] Rettedal E.A., Gumpert H., Sommer M.O.A. (2014). Cultivation-based multiplex phenotyping of human gut microbiota allows targeted recovery of previously uncultured bacteria. Nat. Commun..

[14] Hong B.Y., Lee T.K., Lim S.M., Chang S.W., Park J., Han S.H., Zhu Q., Safavi K.E., Fouad A.F., Kum K.Y. (2013). Microbial analysis in primary and persistent endodontic infections by using pyrosequencing. J. Endod..

[15] Munson M.A., Pitt-Ford T., Chong B., Weightman A., Wade W.G. (2002). Molecular and cultural analysis of the microflora associated with endodontic infections. J. Dent. Res..

[16] WHO (2013). Oral Health Surveys.

[17] Lange D.E., Plagmann H.C., Eenboom A., Promesberger A. (1977). Clinical methods for the objective evaluation of oral hygiene. Dtsch. Zahnarztl. Z..

[18] Garcez A.S., Nuñez S.C., Hamblin M.R., Simões Ribeiro M. (2008). Antimicrobial effects of photodynamic therapy on patients with necrotic pulps and periapical lesion. J. Endod..

[19] Schmittgen T.D., Livak K.J. (2008). Analysing real-time PCR data by comparative Ct method. Nat. Protoc..

[20] Ricucci D., Loghin S., Siqueira J.F. (2013). Exuberant biofilm infection in a lateral canal as the cause of short-term endodontic treatment failure: Report of a case. J. Endod..

[21] Arnold M., Ricucci D., Siqueira J.F. (2013). Infection in a complex network of apical ramifications as the cause of persistent apical periodontitis: A case report. J. Endod..

[22] Ricucci D., Siqueira J.F., Bate A.L., Pitt Ford T.R. (2009). Histologic investigation of root canal-treated teeth with apical periodontitis: A retrospective study from twenty-four patients. J. Endod..

[23] Carr G.B., Schwartz R.S., Schaudinn C., Gorur A., Costerton J.W. (2009). Ultrastructural examination of failed molar retreatment with secondary apical periodontitis: An examination of endodontic biofilms in an endodontic retreatment failure. J. Endod..

[24] Costalonga M., Herzberg M.C. (2014). The oral microbiome and the immunobiology of peridontal disease and carries. Immunol. Lett..

[25] Nóbrega L.M., Montagner F., Ribeiro A.C., Mayer M.A., Gomes B.P. (2016). Bacterial diversity of symptomatic primary endodontic infection by clonal analysis. Braz. Oral Res..

[26] Siqueira J.F., Rôças I.N. (2009). Diversity of endodontic microbiota revisited. J. Dent. Res..

[27] Bouillaguet S., Manoil D., Girard M., Louis J., Gaïa N., Leo S., Schrenzel J., Lazarevic V. (2018). Root microbiota in primary and secondary apical periodontitis. Front. Microbiol..

[28] Tzanetakis G.N., Azcarate-Peril M.A., Zachaki S., Panopoulos P., Kontakiotis E.G., Madianos P.N., Divaris K. (2015). Comparison of bacterial community composition of primary and persistent endodontic infections using pyrosequencing. J. Endod..

[29] Keskin C., Demiryürek E.Ö., Onuk E.E. (2017). Pyrosequencing analysis of cryogenically ground samples from primary and secondary/persistent endodontic infections. J. Endod..

[30] Didilescu A.C., Rusu D., Anghel A., Nica L., Iliescu A., Greabu M., Bancescu G., Stratul S.I. (2012). Investigation of six selected bacterial species in endo-periodontal lesions. Int. Endod. J..

[31] Li H., Guan R., Sun J., Hou B. (2014). Bacteria community study of combined periodontal endodontic lesions using denaturing gradient gel electrophoresis and sequencing analysis. J. Periodontol..

[32] Santos A.L., Siqueira J.F., Rôças I.N., Jesus E.C., Rosado A.S., Tiedje J.M. (2011). Comparing the bacterial diversity of acute and chronic dental root canal infections. PLoS ONE.

[33] Pinheiro E.T., Gomes B.P., Ferraz C.C., Sousa E.L., Teixeira F.B., Souza-Filho F.J. (2003). Microorganisms from canals of root-filled teeth with periapical lesions. Int. Endod. J..

[34] Molander A., Reit C., Dahlén G., Kvist T. (1998). Microbiological status of root-filled teeth with apical periodontitis. Int. Endod. J..

[35] Gomes B.P., Berber V.B., Kokaras A.S., Chen T., Paster B.J. (2015). Microbiomes of endodontic-periodontal lesions before and after chemomechanical preparation. J. Endod..

[36] Özok A.R., Persoon I.F., Huse S.M., Keijser B.J.F., Wesselink P.R., Crielaard W., Zaura E. (2012). Ecology of the microbiome of the infected root canal system: A comparison between apical and coronal root segments. Int. Endod. J..

[37] Manoil D., Al-Manei K., Belibasakis G.N. (2020). A systematic review of the root canal microbiota associated with apical periodontitis: Lessons from next-generation sequencing. Proteom. Clin. Appl..

[38] Rôças I.N., Siqueira J.F. (2012). Characterization of microbiota of root canal treated teeth with post treatment disease. J. Clin. Microbiol..

[39] Gomes B.P., Berber V.B., Kokaras A.S., Chen T., Paster B.J. (2006). Enterococcus faecalis in dental root canals detected by culture and by polymerase chain reaction analysis. Oral Surg. Oral Med. Oral Pathol. Oral Radiol. Endod..

[40] Duque T.M., Prado M., Herrera D.R., Gomes B.P.F.A. (2019). Periodontal and endodontic infectious/inflammatory profile in primary periodontal lesions with secondary endodontic involvement after a calcium hydroxide-based intracanal medication. Clin. Oral Investig..

